# The Interaction of the Metallo-Glycopeptide Anti-Tumour Drug Bleomycin with DNA

**DOI:** 10.3390/ijms19051372

**Published:** 2018-05-04

**Authors:** Vincent Murray, Jon K. Chen, Long H. Chung

**Affiliations:** School of Biotechnology and Biomolecular Sciences, University of New South Wales, Sydney, NSW 2052, Australia; jonken.chen@gmail.com (J.K.C.); chunglonghoa@gmail.com (L.H.C.)

**Keywords:** bleomycin analogues, DNA interaction, double-strand breaks, genome-wide, next-generation sequencing, sequence specificity

## Abstract

The cancer chemotherapeutic drug, bleomycin, is clinically used to treat several neoplasms including testicular and ovarian cancers. Bleomycin is a metallo-glycopeptide antibiotic that requires a transition metal ion, usually Fe(II), for activity. In this review, the properties of bleomycin are examined, especially the interaction of bleomycin with DNA. A Fe(II)-bleomycin complex is capable of DNA cleavage and this process is thought to be the major determinant for the cytotoxicity of bleomycin. The DNA sequence specificity of bleomycin cleavage is found to at 5′-GT* and 5′-GC* dinucleotides (where * indicates the cleaved nucleotide). Using next-generation DNA sequencing, over 200 million double-strand breaks were analysed, and an expanded bleomycin sequence specificity was found to be 5′-RTGT*AY (where R is G or A and Y is T or C) in cellular DNA and 5′-TGT*AT in purified DNA. The different environment of cellular DNA compared to purified DNA was proposed to be responsible for the difference. A number of bleomycin analogues have been examined and their interaction with DNA is also discussed. In particular, the production of bleomycin analogues via genetic manipulation of the modular non-ribosomal peptide synthetases and polyketide synthases in the bleomycin gene cluster is reviewed. The prospects for the synthesis of bleomycin analogues with increased effectiveness as cancer chemotherapeutic agents is also explored.

## 1. Introduction

**Figure 1:**
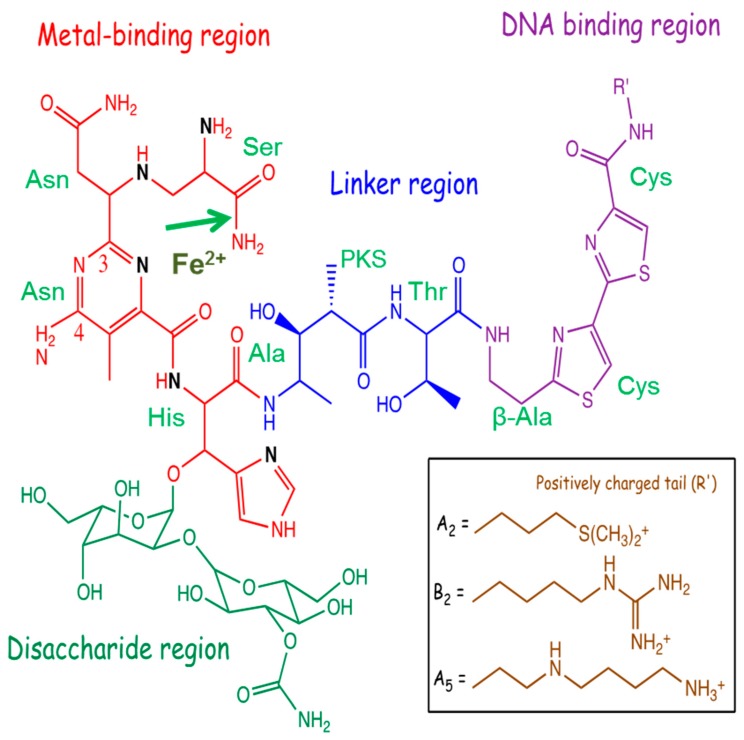
The structure of bleomycin with the four functional domains indicated. The precursor amino acids are shown as well as the propionate moiety derived from polyketide synthases (PKS). The positively charged tails (R′) are shown for the various bleomycin congeners. The green arrow indicates the site of bleomycin hydrolase cleavage. The figure is adapted from [15] and reprinted by permission from Springer Nature.

The bleomycins (Figure 1) are a group of structurally related metallo-glycopeptide antibiotics discovered by Umezama and colleagues [1] and are clinically used as cancer chemotherapeutic agents. The bleomycins are isolated from *Streptomyces verticillis* and the bacterium uses modular non-ribosomal peptide synthetases and polyketide synthases to synthesise bleomycin [2,3,4,5,6]. Bleomycin can be separated by chromatography into bleomycin A and B [1] and both A and B can be further separated into different components. The clinical product, Blenoxane, consists of 60% bleomycin A_2_, 30% bleomycin B_2_ and other minor components. The initial studies showed that bleomycin inhibited DNA synthesis in *E. coli* and HeLa cells and also suppressed the growth of cancer cells including Ehrlich carcinoma and mouse sarcoma [7]. In chemotherapy, bleomycin is currently used in combination with other drugs to treat malignant germ-cell tumours, Hodgkin’s lymphoma and carcinomas of skin, head, and neck [8,9,10,11,12]. In particular, a treatment regimen of bleomycin, etoposide, and cisplatin is able to cure 90% of patients with testicular cancer [11]. Additionally, a combination of bleomycin, vinblastine, and cisplatin have been successfully used to treat metastatic ovarian cancer [13]. Bleomycin’s cytotoxicity is attributed to its ability to cause double- and single-strand DNA breaks, which then lead to extended cell cycle arrest, apoptosis, and mitotic cell death [14,15]. Double-strand breaks are thought to be the most important for anti-tumour activity [16,17,18,19].

## 2. Bleomycin Is Composed of Four Functional Domains

Bleomycin is a relatively large molecule and is composed of four structural domains (Figure 1): a metal-binding region; a linker region; a disaccharide; and a bithiazole tail [20]. Congeners of the bleomycin family are differentiated by the positively charged region on the bithiazole tail.

The metal binding region contains nitrogen atoms that are involved in coordination with transition metals [21,22,23,24]. Bleomycin is able to coordinate with a number of metals including: Fe, Co, Cu, Mn, V, and Zn. Fe ions are thought to be the biologically important divalent cations that are responsible for the cytotoxic activity of bleomycin. The metal-binding region is also thought to play an important role in the DNA sequence specificity of bleomycin. Structural studies of Co(III)-bleomycin bound to DNA show the interaction of the N_3_ and C_4_-NH_2_ of the bleomycin pyrimidine moiety with the C_2_-NH_2_ and N_3_ of guanine [20].

In conjunction with the metal-binding and linker region, the C-terminal bithiazole tail is important for DNA recognition and binding of bleomycin with DNA. The bithiazole tail and its C- terminal region is thought to contribute to DNA binding by intercalation or minor groove interaction [15,25,26]. With a positive charge on the R′-group, this tail facilitates the electrostatic interaction between bleomycin and DNA. Drugs with a planar fused ring system can interact with DNA via intercalation and hence it is expected that the coplanar bithiazole tail of bleomycin can intercalate between bases of the DNA to increase DNA affinity [23,27]. However, other studies pointed out that bleomycin might interact with DNA via minor grove binding [25,26,28]. More recent research using X-ray crystallography indicates that intercalative binding is the main form of DNA binding (see below) [15,20].

The function of the disaccharide region of bleomycin is not thoroughly understood because of the lack of analogues with varying sugar groups [29]. The disaccharide moiety consists of an l-gulose and 3-*O*-carbamoyl-d-mannose. A modified bleomycin complex, deglycobleomycin, where the disaccharide moiety is replaced with hydrogen, demonstrated a reduced efficiency of DNA cleavage [30]. Studies by the Hecht group have highlighted the role of the disaccharide moiety in the selectivity and uptake of bleomycin by tumour cells [31]. They demonstrated that the bleomycin disaccharide alone was sufficient for selective uptake by tumour cells [32,33,34]. The 3-*O*-carbamoyl-d-mannose moiety appears to be crucial for this function [35,36]. This is consistent with related work that provided evidence that this sugar region is involved in tumour cell uptake via glucose transport. Glycolysis is accelerated in tumour cells due to their high energy demands. Bleomycin may be effective in targeting cancer cells because the drug can be mistaken as glucose due to its disaccharide moiety and hence, freely enter into tumour cells via protein channels [33,34].

The linker region plays a role as a bridge that joins the metal binding and DNA binding domains [37]. The linker connects the metal binding region and bithiazole tail; and consists of a valerate and l-threonine subunit. Systematic modifications to the substituents of these subunits revealed their importance for the efficiency of bleomycin-mediated DNA cleavage [29,38,39,40]. Notably, these analogues exhibit significant reduction in DNA cleavage (compared to deglycobleomycin) but maintained a similar sequence specificity. On the other hand, analogues methylated at the valerate-threonine amide were reported to have diminished sequence specificity [41]. These studies suggest the importance of the linker in inducing an optimal conformation of bleomycin with respect to DNA binding. The X-ray crystal structure revealed that the valerate was hydrogen bonded to the minor groove of DNA [20].

## 3. Bleomycin DNA Cleavage Mechanism

When bleomycin, in its metal-free form, is administrated intravenously, it rapidly binds to Cu(II) in the blood serum to create a stable complex, bleomycin-Cu(II) [23]. When this complex is transferred inside a cell, bleomycin is reduced to bleomycin-Cu(I). The new complex can enter the nucleus and exchange with Fe(II) to form bleomycin-Fe(II).

The degradation of DNA by bleomycin is preceded by the formation of an activated form of bleomycin [15]. Activated bleomycin has been shown to form in the presence of O_2_, when bleomycin-Fe(II) binds to O_2_, forming bleomycin-Fe(II)-OO*^•^*, which then accepts an electron and H^+^ to generate the “activated bleomycin” complex, bleomycin-Fe(III)-OOH. Regardless of the presence of DNA, the activated bleomycin complex decays rapidly, with a half-life of approximately 2 min at 4 °C and ultimately forms bleomycin-Fe(III) [42].

**Figure 2:**
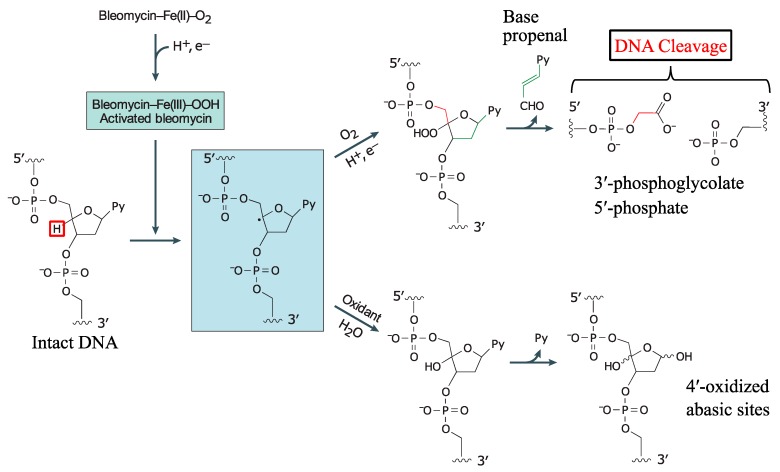
Mechanism of bleomycin-mediated DNA cleavage. The Bleomycin-Fe(III)-OOH activated form is generated in the presence of a one-electron reductant, Fe^2+^ and oxygen. The activated bleomycin then abstracts the hydrogen atom (red square) from C4′ of the deoxyribose moiety of DNA to form the intermediate 4′ radical. This intermediate can partition into two pathways. In the abundance of oxygen, the 4′ radical initiates a series of chemical transformations, leading to a direct strand break and producing 3′-phosphoglycolate and 5′-phosphate ends, and release of a base propenal. However, in the absence of oxygen, the intermediate reacts with an oxidant in the presence of water, generating 4′-oxidized abasic sites. The figure is adapted from [15] and reprinted by permission from Springer Nature.

Activated bleomycin participates in the abstraction of the C4′ hydrogen atom from the deoxyribose moiety of a pyrimidine nucleotide 3′- to a guanine (Figure 2) [23,28,43]. This results in the formation of a C4′ radical intermediate that can proceed via two separate pathways, depending on the presence of O_2_. In the absence of O_2_, 98% of the C4′ radical is converted to 4′-oxidised abasic sites [44]. On the other hand, in the presence of 1 atmosphere of O_2_, approximately 70% undergoes strand scission and forms 3′-phosphoglycolate and 5′-phosphate ends, while the remainder forms 4′-oxidised abasic sites [45,46].

In the oxygen-dependent pathway, the C4′ radical reacts with O_2_ to form a peroxyl radical, which is then reduced to a 4′-hydroperoxide (Figure 2). The latter undergoes further chemical transformations that ultimately leads to DNA strand scission, releasing a base propenal. The resulting gap comprises 3′-phosphoglycolate and 5′-phosphate terminal ends. Alternatively, the C4′ radical can also initiate an oxygen-independent pathway where the C4′ radical undergoes oxidation to form a 4′-carbocation that is subsequently hydroxylated and finally generates a C4′-oxidised abasic site. The 4′-oxidised abasic site is unstable, with a half-life of 8–26 h at pH 7; it is also alkali labile and capable of undergoing β-elimination that results in DNA strand scission, forming a phosphate and 4′-ketodeoxyribose at the 5′- and 3′-ends, respectively [46,47].

Activated bleomycin has also been reported to catalyse the degradation of other cellular components including RNA [48], lipids [49], and proteins [50]. Cleavage of RNA by bleomycin was shown to be selective, depending on the secondary and tertiary structure of the substrate [51,52]. The significance of these bleomycin cellular targets is not fully understood since the cytotoxic nature of bleomycin is thought to mainly stem from its ability to mediate double-strand DNA cleavage [16,17,18,19].

## 4. Bleomycin Cleavage Specificity with Purified DNA

With the addition of Fe^2+^, the cleavage of purified DNA by bleomycin can be observed. Using defined sequences of purified DNA, the DNA sequence specificity of bleomycin cleavage can be determined. Bleomycin preferentially cleaves DNA at particular dinucleotide sequences. DNA sequence specificity studies with purified plasmid sequences have indicated that the dinucleotides 5′-GT* and 5′-GC* are preferentially cleaved by bleomycin [53,54,55,56,57,58,59,60,61,62,63,64,65,66,67,68]. For ease of discussion in this review, the bleomycin cleavage site nucleotides are numbered with respect to the cleavage site at nucleotide position 0, and the positions discussed are from −3 to +2 (see Table 1). The * indicates the nucleotide at position 0 that is cleaved and destroyed during the reaction. Bleomycin has also been observed to cleave, to a lesser extent, at the dinucleotides GG*, GA*, AT*, AC*, and AA* [58,59].

Early studies noted that the relative intensity of cleavage between different sites with the same dinucleotide (e.g., 5′-GT*) can vary, suggesting that the DNA sequence determinant of the specificity of bleomycin cleavage is not confined to just two nucleotides [57,58]. Murray et al. found that the nucleotide immediately 5′ to the bleomycin dinucleotide cleavage site (position −2) affected the cleavage intensity; an adjacent thymine enhanced, whereas guanine and adenine reduced the cleavage intensity [58,59]. It was also observed that alternating purine-pyrimidine sequences were preferred cleavage sites for bleomycin [59].

Hecht’s group utilised hairpin DNA containing a motif with a randomised sequence to identify variations that are strongly bound by bleomycin [69]. These hairpins consisted of single-stranded DNA of 64 nucleotides in length and when self-annealed, the hairpin DNA contained an 8 bp double-stranded motif with a randomised sequence at the centre. A mixture of these randomised hairpin DNAs was then incubated with resin-bound bleomycin and variations that strongly bound to bleomycin were isolated and sequenced. From this, they identified hairpin variations that were shown to strongly inhibit bleomycin cleavage of a 5′-GC-3′-containing hairpin in a competition assay [63,70]. It is interesting to note that among these hairpin variations, some did not contain any 5′-GT* or 5′-GC* cleavage sites and yet they exhibited strong competition against the assay hairpin. They also examined the sequence specificity of bleomycin cleavage with these hairpins. While the strongest cleavage was observed at the canonical 5′-GT* and 5′-GC* sites, they also reported efficient cleavage at non-conventional sites containing AT-rich dinucleotides [63,71]. Later studies reported that hairpins with a 5′-GCGT sequence bound strongly to bleomycin and produced high intensity double-strand breaks [66]. It is not clear whether the hairpin structures that were used in these experiments had unusual DNA microstructure that influenced the results.

## 5. Bleomycin Cleavage Specificity with Purified DNA Using Updated Technology

More recently, updated technology using capillary electrophoresis with laser-induced fluorescence (CE-LIF) and automated DNA sequencers, has permitted a more accurate and precise determination of the DNA sequence specificity of bleomycin DNA damage in longer purified DNA sequences [72,73,74,75,76,77,78,79]. In addition, the use of separate 5′- and 3′-end-labelling in a DNA sequence specificity study contributes to the precision of the process by greatly reducing end-label bias [78,79]. It should be noted that experiments with end-labelled DNA mainly detect single-strand breaks (see below).

Using these techniques, a more detailed bleomycin sequence specificity was elucidated using two random DNA sequence of 247 and 425 bp in length [78]. For a random DNA sequence to contain all the possible sequences, it must be sufficiently long. For a four bp recognition sequence, it must be at least 256 bp and for a five bp recognition sequence, it must be at least 1024 bp in length. Hence, for the 247 and 425 bp sequences, a 4 bp recognition sequence could be extracted. It was found that bleomycin preferentially cleaved at 5′-TGT*A DNA sequences [78].

Our genome-wide studies (see below) indicated that in human cells, the DNA sequence 5′-RTGT*AY (where R is G or A, and Y is T or C) was preferentially cleaved by bleomycin [80]. This 5′-RTGT*AY sequence was systematically altered by varying these nucleotides in a cloned DNA sequence. The bleomycin cleavage efficiency was then investigated in these variant nucleotide sequences. This study permitted the importance of flanking nucleotides around the 5′-GT, 5′-GT*A and 5′-TGT*A core sequences to be evaluated. It was observed that the preferred nucleotide sequence for high intensity bleomycin cleavage was 5′-YYGT*AW (where W is A or T) (Table 1). The DNA sequence that had the highest intensity of bleomycin cleavage was 5′-TCGT*AT and the seven highest intensity bleomycin cleavage sites conformed to the 5′-YYGT*AW consensus sequence [79]. This study permitted a precise evaluation of crucial neighbouring nucleotides that produced high intensity bleomycin DNA cleavage sites. This approach of systematically altering the nucleotides around bleomycin cleavage sites is a powerful method of analysis because every possible nucleotide is included in the analysis and no variant is omitted [79]. This is in contrast to an analysis of random DNA sequences where some nucleotide variants may not be present in the analysis. This study confirmed that 5′-GT, 5′-GT*A, and 5′-YGT*A were core sequences for high intensity bleomycin cleavage sites.

**Figure 3:**
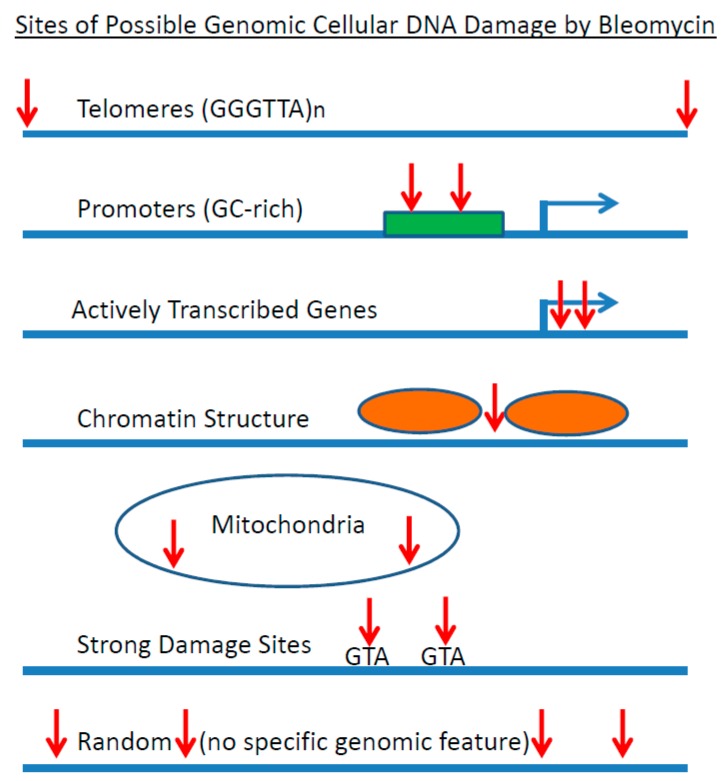
Sites of possible genomic cellular DNA damage by Bleomycin. Red arrows indicate bleomycin cleavage sites, while blue arrows are transcription start sites. The green rectangle is a promoter and the orange ovals are nucleosome cores.

Human telomeres are composed of tandem repeats of the DNA sequence 5′-GGGTTA. Seventeen tandem repeats of this sequence were cloned into a plasmid DNA and the bleomycin sequence specificity was investigated [72,75]. It was found that bleomycin preferentially cleaved at 5′-GT dinucleotides in the telomeric sequence. Hence, telomeric DNA sequences are an important genomic site for bleomycin cleavage (Figure 3).

## 6. Sequence Specificity at Bleomycin-Induced Abasic Sites

As well as cleaving DNA, bleomycin also produces abasic sites in DNA. The enzyme endonuclease IV is able to cleave DNA at abasic sites. Hence endonuclease IV can be used to examine the sequence specificity of bleomycin abasic site damage. It was found that bleomycin abasic DNA damage preferentially occurs at 5′-GC and 5′-GT sites compared with the 5′-GT preference observed for bleomycin-induced phosphodiester strand breaks [77].

Abasic DNA damage is enhanced in the absence of molecular oxygen and tumours are thought to be hypoxic [81,82]. Hence, the sequence specificity of bleomycin-induced abasic DNA damage is important for the effectiveness of anti-tumour agents and should be taken into account during the design of bleomycin analogues [2,29].

## 7. X-ray Crystal Structure of Bleomycin with DNA

An X-ray crystal structure of bleomycin bound to DNA has been determined [20]. In this structure, a Co(III)-bleomycin B_2_ was bound to a DNA sequence containing a 5′-GT. It was shown that the bithiazole moiety intercalated into DNA. The metal binding and disaccharide domains were bound in the minor groove of DNA utilising hydrogen bonding. The Co(III) metal coordination involved the primary amine of β-aminoalanine as an axial ligand; and the secondary amine of β-aminoalanine, imidazole, histidine amide, and pyrimidine N1 as equatorial ligands. A modelled hydroperoxide ligand coordinated to Co(III) was perfectly positioned for abstraction of a hydrogen atom from C4′-H. A caveat about this X-ray crystal structure was that Co(III) was used instead of the physiologically important Fe(II).

An important feature of the X-ray crystal structure was that the bithiazole moiety was shown to be intercalated into DNA; whereas previously, minor groove binding of the bithiazole group was suggested [25,26,28]. The linker methylvalerate OH was found to be involved in hydrogen bonding to DNA. The NH_2_ of 3-*O*-carbamoyl-d-mannose was also shown to hydrogen bond with DNA in the minor groove; whereas no function could be discerned for the L-gulose since it was not involved in DNA binding.

Concerning the sequence specific interaction of the Co(III)-bleomycin B_2_ complex with DNA, a number of hydrogen bonds were made between the bleomycin complex and the minor groove of DNA. With reference to the 5′-TAGT*TAAC sequence used in the experiments, there were three hydrogen bonds with the crucial G nucleotide at position −1, one at position −2, and one at position −3. This explains the importance of the G nucleotide at position −1, but also shows that an extended bleomycin sequence specificity could be due to hydrogen bonding at positions upstream and downstream from the dinucleotide cleavage site. In addition, the hydroperoxide ligand was positioned close to the C4′-H of the T nucleotide at position 0 that would result in the cleavage of DNA at this T nucleotide.

The bleomycin complex was bound to a 5 bp section of DNA. The bithiazole group was intercalated between the 5′-T*T dinucleotide (positions 0 and +1); and the intercalation unwound the double helix 3′ to the 5′-GT* dinucleotide (positions −1 and 0). These features have implications for the DNA sequence specificity of bleomycin (see below).

## 8. Mechanism of Bleomycin-Induced Double-Strand Break Formation

Double-strand breaks are primarily derived from single-strand breaks and the ratio of double-strand to single-strand breaks has been reported to be in the range 1:3 to 1:20 [16,27,83,84,85,86]. Both single-strand and double-strand break pathways share the common intermediate of a 4′-radical; however, the double-strand break process strictly occurs in the presence of oxygen [86,87,88]. A bleomycin double-strand cleavage event generates new DNA fragments with either blunt or 5′-staggered ends depending on the recognition sequences. A double-strand cleavage model proposed by Steighner and Povirk suggested that a single molecule of bleomycin carried out the cleavage events on both strands of DNA [84,86,87,89]. After cleaving at one site designated as the primary site, the molecule must be reactivated and swiftly relocate itself to the secondary site on the other strand to make another cut [84]. As a result, the ratio of double-strand to single-strand breaks is dependent on the likelihood of bleomycin reactivation after the primary cleavage, on the rate of molecular relocation and reorganisation, as well as the DNA sequences present. During the relocation, the DNA-binding domain (the bithiazole tail) plays a role in maintaining strong binding with the DNA so that the molecule is able to relocate itself to the secondary cleavage site without dissociating from the DNA. Therefore, a good primary cleavage site is more likely to give rise to the secondary cleavage site because in this case the bleomycin molecule has a strong interaction with DNA from its primary contact and its dissociation is low [89]. Through structural studies of bleomycin-Co(II)-OOH with 2D NMR, the mechanism of this rearrangement was proposed to involve the rotation of the partially intercalated bleomycin molecule after the primary cleavage event [23,88,90]. Acting together with the tail, the linker region acts as a tether to bring over the cleaving metal-binding domain to the secondary cleavage site. This might explain why modifying the linker region not only affects the efficiency of DNA cleavage but also reduces the double-strand to single-strand break ratio [91,92].

Studies with short sequences (500 bp) with a small number of sites (100 cleavage sites) have been used to detect and quantify double-strand breaks. It was proposed that a double-strand break primarily comes from a strong primary site which is 5′-GY* (5′-GT* or 5′-GC*) and the secondary cleavage site is determined by the nucleotide that is 3′- to the Y nucleotide. If this 3′-adjacent nucleotide is a pyrimidine, then the second cleavage will generate blunt-end fragments. In contrast, if it is a purine, then 5′-staggered-end fragments are produced [27,83,84,86,87,89]. Their studies also found that the palindromic site 5′-GT*AC was a hotspot for double-strand cleavage. 

It was also observed that, subsequent to the cleavage of the primary site, the secondary site can partition into the formation of either a strand scission, containing the 3′-phosphoglycolate and 5′-phosphate ends; or a 4′-oxidised abasic site. In contrast, the primary cleavage site was always observed to form 3′-phosphoglycolates at the double-strand breaks [84]. Additionally, Absalon et al. reported that bleomycin-induced double-strand breaks do not form under oxygen-depleted conditions and this further supports the observation that the primary cleavage site must be a bleomycin-induced strand scission [87].

The single-strand to double-strand cleavage ratio of bleomycin was found to be conserved over a large range of bleomycin concentrations [83,86]. This observation is not consistent with the theory that the formation of double-strand breaks arises via the accumulation of random and independent single-strand cleavages, where one would expect the single-strand to double-strand break ratio to decrease when the concentration of bleomycin is increased. These findings have led to the proposal of a model which involves the reactivation of a single bleomycin to produce a double-strand break via cleavage on both strands.

## 9. The Sequence Specificity in Intact Human Cells

Bleomycin DNA damage has also been examined in intact human cells [93]. The bleomycin DNA sequence specificity was determined in human cells with repetitive centromeric alphoid DNA sequences [93], telomeric DNA sequences [74], globin [94,95,96], and retinoblastoma genes [97], with the DNA sequence specificity again found to be concentrated at the dinucleotides 5′-GT* and 5′-GC* in human cells. For the globin and retinoblastoma DNA sequences, bleomycin was able to footprint transcription factors and positioned nucleosomes in human cells. Bleomycin is known to cleave in the linker region of nucleosomes [93,98,99] and hence bleomycin is also a useful agent to probe chromatin structure in human cells (Figure 3).

Human telomeric sequences are composed of the tandemly repeated DNA sequence (GGGTTA)_n_. Since the 5′-GT* dinucleotide is a main site for bleomycin cleavage, telomeric sequences are expected to be a major site for bleomycin cleavage (Figure 3). This was found to be the case where bleomycin preferentially cleaved at telomeric 5′-GT* dinucleotides in human cells [74]. Since telomeres are important in chromosome replication, it has been hypothesised that telomeres could be a crucial genomic site for the cytotoxicity of bleomycin [74,75].

## 10. Sequence Specificity of Bleomycin Double-Strand Breaks in the Entire Human Genome

In our laboratory, the sequence specificity of bleomycin-induced double-strand breaks in the entire human genome has been studied by use of massively parallel next-generation sequencing technology. Contrary to the approximately 100 sites investigated with the previous techniques, the next-generation sequencing technology has enabled more than 200 million bleomycin double-strand break sites in both cellular and purified DNA to be assessed in order to determine the genome-wide sequence specificity [80,99,100,101]. It should be noted that the genome-wide studies mainly detect double-strand breaks, whereas the experiments with purified end-labelled DNA sequences (discussed above) mainly detect single-strand breaks.

The genome-wide study calculated the frequency of occurrence of dinucleotide, trinucleotide, tetranucleotide, pentanucleotide, and hexanucleotide DNA sequences at bleomycin cleavage sites as well as the nucleotide frequency at each position for the ten nucleotides 5′ to the cleavage site and eleven nucleotides 3′ to the cleavage site. These analyses were performed for the 50,000 highest intensity cleavage sites to reveal the genome-wide DNA sequence specificity of bleomycin in human cells. This genome-wide method gave a longer preferred bleomycin cleavage site DNA sequence specificity than previous methods. For the 50,000 highest intensity cleavage sites, the preferred bleomycin cleavage sites were at 5′-GT* dinucleotide sequences, 5′-GT*A and 5′-TGT* trinucleotide sequences, 5′-TGTA tetranucleotide sequences and 5′-ATGT*A pentanucleotide sequences. For cellular DNA, the hexanucleotide DNA sequence 5′-RTGT*AY was the most highly cleaved DNA sequence. This finding strongly agreed with the observation that alternating purine-pyrimidine sequences are preferentially cleaved by bleomycin [78,80]. The core bleomycin cleavage site is probably the 5′-TGT*A tetranucleotide sequence since it was more frequently cleaved by about three-fold compared to the next-ranked tetranucleotide sequence.

There were differences between the genome-wide DNA sequence specificity of bleomycin cleavage in purified DNA compared with cellular DNA. These differences mainly occurred at sequences flanking the core 5′-TGT*A tetranucleotide cleavage sequence. With cellular DNA, the most highly cleaved sequence was the hexanucleotide 5′-RTGT*AY; whereas it was the pentanucleotide 5′-TGT*AT for purified DNA (Table 1). 

There are two methods for analysing the genome-wide DNA sequence specificity of bleomycin cleavage. In the first method, the entire sequence present at the bleomycin cleavage site is examined; this is the method of analysis discussed in the previous paragraph and involves dinucleotides, trinucleotides, tetranucleotides, pentanucleotides, and hexanucleotides. In the second method, the individual nucleotides present at the bleomycin cleavage site are examined by frequency calculations. Using the second method, the statistically preferred nucleotides for cellular DNA were GTGT*A; whereas it was TGT*AW for purified human DNA. Again, the core sequence was the same, 5′-TGT*A, but there were variations in the flanking sequences at positions −3 and +2 (Table 1).

However, the first method of analysis (the complete sequence) gives probably the most accurate representation of the sequence specificity of bleomycin DNA cleavage since the second method suffers from the drawback that the nucleotides at the bleomycin cleavage site may not exist together at the actual cleavage sequence [80].

## 11. Comparison of the Bleomycin Genome-Wide DNA Sequence Specificity with Purified Plasmid DNA Sequences

The genome-wide bleomycin DNA sequence specificity was compared to results obtained from end-labelled purified plasmid DNA sequences. By examining the bleomycin sequence specificity in a cloned section of human DNA, a comparison could be made between a DNA sequence in purified DNA and the identical sequence in the genome-wide data. Human mitochondrial DNA sequences were utilised for this task after cloning into plasmids. Two sections of human mitochondrial DNA were investigated, and it was found that at individual bleomycin cleavage sites, there was a very low level of correlation in the intensity of bleomycin cleavage in the two environments [78]. However, at an overall level the bleomycin sequence specificity, 5′-TGT*A, was similar in the two environments.

As described above, the sequence in the human genome that was preferentially cleaved by bleomycin was found to be 5′-RTGT*AY [80]. This sequence along with systematically altered nucleotide variations, was placed into a plasmid construct called the RTGT*AY plasmid. The consensus DNA sequence derived from the most highly cleaved bleomycin cleavage sites with this plasmid was 5′-YYGT*AW [79]; while from the genome-wide data, it was 5′-TGT*AW for purified genomic DNA from the individual nucleotide data; and 5′-TGT*AT for purified genomic DNA from the complete sequence data (Table 1). There was a consistent core of 5′-GT*A in these consensus sequences. At the −3 position, it was C > T for the plasmid, and no statistically significant nucleotide preference for the purified genome-wide (Table 1). At the −2 position, it was C = T for the plasmid, and T for the purified genome-wide. At the +2 position, it was T = A for the plasmid, and T > A for the purified genome-wide.

The differences between the plasmid and the purified genome-wide can probably be attributed to the techniques used to obtain the data; the end-labelled plasmid data detected single-strand breaks, while purified genome-wide detected double-strand breaks. A similar process is thought to occur in the production of these breaks, but a double-strand break is thought to be a more extreme event [15,84].

During the genome-wide procedure, double-strand breaks are ligated to linkers before being added to the Illumina flowcell. This ligation procedure may not be sequence independent and may introduce sequence bias into the results; whereas the CE-LIF end-labelling procedure is simpler and has fewer steps.

The main drawback of the end-labelling procedure is that only a small number of DNA sequence sites can be examined; whereas hundreds of millions of double-strand breaks can be examined in the genome-wide procedure. In addition, the sequences in the genome-wide experiments are essentially random; whereas the sequence composition is constrained in plasmid constructs.

## 12. Comparison of the Bleomycin Genome-Wide Sequence Specificity in Cellular DNA Compared with Purified Genomic DNA Sequences

There were differences between the genome-wide DNA sequence specificity of bleomycin in purified DNA compared with cellular DNA. Both sets of data were derived from the Illumina system and therefore double-strand breaks were detected for both types of DNA environments.

The consensus DNA sequence derived from the most highly cleaved bleomycin cleavage sites was 5′-TGT*AW for purified genomic DNA and 5′-GTGT*A for cellular DNA from the individual nucleotide data; and 5′-TGT*AT for purified genomic DNA and 5′-RTGT*AY for cellular DNA from the complete sequence data (Table 1). The main differences were at the −3 and +2 positions since the core 5′-TGT*A was the same for the two environments. At the −3 position, no statistically significant nucleotide preference was found for the purified genome-wide, and G or R for the cellular genome-wide (Table 1). At the +2 position, it was T > A for the purified genome-wide and no nucleotide preference or Y for the cellular genome-wide.

The environment of cellular DNA is different to purified DNA in a number of ways. Cellular DNA is complexed with proteins and DNA-bound cellular proteins have a large influence on the interaction of bleomycin with cellular DNA. Bleomycin is a relatively large molecule (1500 daltons) and has difficulty accessing DNA bound to proteins. Cellular DNA is mainly found complexed with histones in the form of nucleosomes. Nucleosome cores are known to protect DNA from bleomycin cleavage [98,102]. DNA binding proteins, for example transcription factors, have also been observed to protect DNA from bleomycin cleavage [94,95,96,97,102]. Proteins bound to DNA can distort the structure of DNA and this can lead to an alteration in the DNA sequence specificity of bleomycin cleavage in cellular DNA compared to purified genomic DNA. In addition, cellular DNA is supercoiled and hence has a distorted DNA structure that could also lead to changes in bleomycin sequence specificity.

DNA is also a dynamic molecule in cells where DNA replication and transcription produce transient single-stranded regions that will result in an altered bleomycin interaction. The cellular environment contains many different chemical constituents and the cation bound to bleomycin can vary inside cells where not all of the Cu(I)- or Cu(II)-bleomycin may be exchanged with Fe(II); whereas in the purified experiment, the metal cation bound to bleomycin can be completely controlled.

## 13. Conformation of DNA and the DNA Sequence Specificity of Bleomycin

The DNA microstructure is likely to be very important for the intensity of bleomycin cleavage at each lesion site. The conformation of DNA is derived from the DNA sequence where the order of bases has a major influence on the DNA microstructure [103,104,105,106]. Hence, the consensus DNA sequence that is found from the DNA sequence specificity experiments will give rise to a particular DNA structure that is highly conducive to bleomycin cleavage; while other DNA sequences will have a different DNA structure that is less productive for bleomycin cleavage. The features of DNA structure that are likely to be important for bleomycin binding and cleavage are: the intercalation of the bithiazole group into the DNA helix; the productive binding in the minor groove by the metal binding, linker, and disaccharide domains; and the positioning of the complex to abstract a hydrogen atom from C4′-H. If these major features are optimally present, then a high intensity bleomycin cleavage site is likely to occur.

The other important parameter is the interactions of the bleomycin molecule with specific nucleotides in DNA. These interactions were revealed by the X-ray crystal structure of bleomycin with DNA and indicated that hydrogen bonding between the bleomycin complex and particular nucleotides, especially the G nucleotide at position −1, in the minor groove of DNA were important. Hence, a combination of specific DNA sequence interactions and the microstructure of DNA are likely to be the main determinant of the DNA sequence specificity of bleomycin. Of course, the DNA sequence informs the microstructure of DNA and the two are interrelated.

The X-ray crystal structure found that the bleomycin complex was bound to a 5 bp section of DNA [20]. This is consistent with the pentanucleotide and hexanucleotide consensus sequences presented above for the DNA sequence specificity of bleomycin. The bithiazole group was intercalated between the dinucleotide at positions 0 and +1.

The binding of proteins to DNA and other cellular parameters will also alter the DNA conformation and lead to differences in bleomycin DNA cleavage in cells compared to purified DNA.

## 14. Chromatin Structure Affects the Interaction of Bleomycin with Cellular DNA

As mentioned above, chromatin structure affects the interaction of bleomycin with cellular DNA where nucleosome cores and other DNA binding proteins prevent bleomycin from cleaving DNA [102]. It has been demonstrated that bleomycin targets the linker region of nucleosomes rather than the core DNA and it can be used to footprint chromatin structure in human cells [98,107,108].

**Figure 4:**
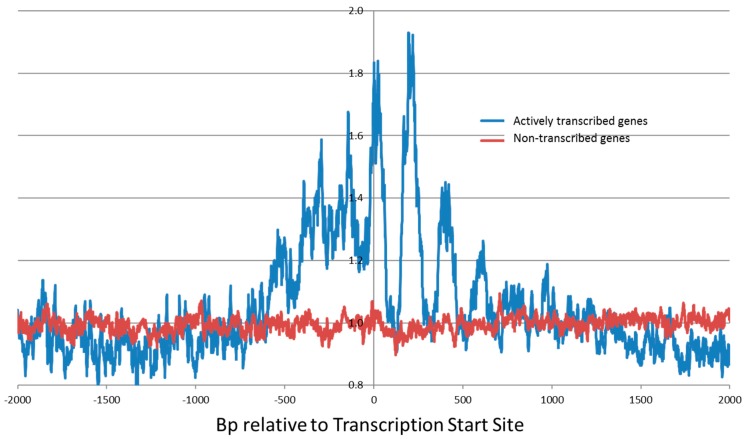
Bleomycin damage at Transcription Start Sites (TSSs) for actively transcribed genes (**blue**) and non-transcribed genes (**red**) in human HeLa cells. The cellular/purified DNA ratio is on the *y*-axis and this ratio removed any bias due to the DNA sequence preference of bleomycin cleavage. These data were compiled from the 24,402 most highly-expressed genes in HeLa cells and 82,596 non-transcribed genes in HeLa cells [99]. Note that the spacing between peaks is approximately 200 bps and probably corresponds to phased nucleosomes in these transcribed regions. The *x*-axis is the position of DNA cleavage at each nucleotide (bp) relative to the TSS. Reprinted by permission from Springer Nature [99].

Our genome-wide Illumina next-generation sequencing studies revealed an enhanced cleavage pattern for bleomycin at transcription start sites (Figure 4). The peaks of the enhanced bleomycin cleavage were approximately 200 bp apart. This implies that positioned nucleosomes are present at transcription start sites and that bleomycin preferentially cleaves in the linker region of the nucleosome and the nucleosome core protects DNA from bleomycin cleavage [109,110,111]. Hence, bleomycin can be used to detect chromatin structure at actively transcribed genes [99].

Bleomycin preferentially damaged actively transcribed genes (Figure 4) and the degree of bleomycin cleavage correlated with the level of transcription [99]. This enhanced bleomycin damage occurred within 1000 bp of transcription start sites (TSSs). The 143,600 identified human TSSs were split into non-transcribed genes (82,596) and transcribed genes (61,004) for HeLa cells. The bleomycin cleavage pattern at highly transcribed gene TSSs was greatly enhanced compared with non-transcribed gene TSSs. Genes that are actively being transcribed have a more open chromatin structure compared with non-transcribed genes; hence bleomycin will be able to cleave at these active genes compared with the more closed chromatin structure of non-transcribed genes. There were also differences that depended on whether the sense or antisense strand were analysed.

For individual genes, the degree of bleomycin cleavage at TSSs was also determined and a ratio of cellular/purified DNA cleavage was calculated. As expected, it was found that highly transcribed genes had a higher cellular/purified ratio than non-expressed genes. In particular the following genes had a high cellular/purified ratio: SERF2, TM4SF1, and TUBA1B (tubulin). Targeting of actively transcribed genes in conjunction with bleomycin could enhance the cancer chemotherapeutic efficacy of bleomycin [112,113]. In combination with nucleic acid-based techniques that target these crucial genes, bleomycin cytotoxicity could be increased by focusing on these important genes.

## 15. Cancer Signal Transduction Pathways Affected by Bleomycin

It has been estimated for bleomycin that 500 double-strand breaks or 150,000 single-strand breaks were required to induce apoptosis in Chinese hamster fibroblasts [19]. Bleomycin appeared to mediate two types of cell death, depending on the intracellular accumulation of the drug. By using electropermeabilisation, Tounekti et al. could control the amount of bleomycin that is introduced into DC-3F Chinese hamster fibroblasts and the human head and neck carcinoma cell line A-253 [114]. At low cellular concentrations, cells were observed to enter cell cycle arrest at the G2-M phase that eventually led to mitotic death. On the other hand, at high concentrations, bleomycin was proposed to act as a micronuclease since it was observed to rapidly cause significant fragmentation to the DNA and induced an apoptosis-like pathway.

Treatment of cells with DNA-damaging agents, such as bleomycin, is associated with the activation of p53 [115,116]. Subsequently, the activated p53 can mediate apoptosis via the regulation of the Bcl-2 protein family and the release of cytochrome c from the mitochondria. For example, bleomycin treatment of HEp-2 cells resulted in apoptosis via the activation of p53 and a conformational change of Bax, coupled with the release of cytochrome c and the apoptosis-inducing factor [116].

Bleomycin has been observed to induce p53- and Bcl-2-independent apoptosis in squamous carcinoma cells [117]. Furthermore, p53-deficient HL-60 cells were able to induce mitochondria-mediated apoptosis [118]. Alternatively, the apoptosis-inducing factor promotes apoptosis via a caspase-independent cell death. The release of the apoptosis-inducing factor and the DNase endonuclease G from the mitochondria, and subsequent translocation into the nucleus, causes large-scale DNA fragmentation [119,120].

**Figure 5:**
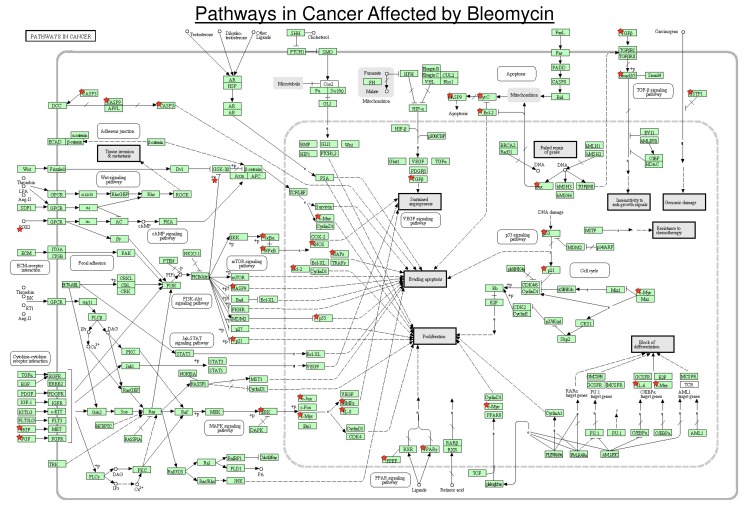
Pathways in Cancer affected by Bleomycin. In order to determine the reactions, genes and gene products that were affected by bleomycin, the Comparative Toxicogenomics Database (Available online: http://ctdbase.org/) was utilised with a requirement of a minimum of three hits. The Database for Annotation, Visualization, and Integrated Discovery (DAVID) tool (Available online: http://david.abcc.ncifcrf.gov/home.jsp) was then used to map these genes onto the Kyoto Encyclopedia of Genes and Genomes (KEGG) Pathways in Cancer map [121]. A step in the Pathways in Cancer KEGG map that was affected by bleomycin is indicated by a red star.

Bleomycin has a major effect on a number of signal transduction pathways. With regard to the signal transduction pathways involved in cancer, we utilised several tools to visualise the effect of bleomycin on these pathways. Using the Comparative Toxicogenomics Database and the Database for Annotation, Visualization, and Integrated Discovery tools, the bleomycin-affected genes were mapped onto the Kyoto Encyclopedia of Genes and Genomes (KEGG) Pathways in Cancer (Figure 5) [121]. The red stars in Figure 5 indicate the proteins/genes that were affected by bleomycin and it reveals that bleomycin impacts a number of signal transduction pathways involved in cancer. These include the PI3K-Akt, MAPK, p53, PPAR, and other pathways. This results in changes to caspases in apoptotic pathways; changes in proliferation, repair, DNA damage response, cell cycle, angiogenesis, evading apoptosis, differentiation, and insensitivity to anti-growth signals.

Note that the signal transduction pathways in cancer are highly complex and not fully understood; but the important point revealed in Figure 5 is that bleomycin acts at a number of these pathways and this could provide an explanation for the effectiveness of bleomycin as a cancer chemotherapeutic agent. Instead of affecting a single section of one pathway or a single crucial modification site, bleomycin impacts on a number of signal transduction pathways. Hence, it is more difficult for a tumour cell to develop resistance to these multiple blocks in signal transduction pathways compared with a single blockage that could be overcome by a single mutation/modification. In order to develop resistance to the multiple blocks caused by bleomycin, a number of resistance mechanisms must be generated simultaneously which is much harder than for a single block [121,122,123,124].

## 16. Repair of Bleomycin-Induced DNA Damage

### 16.1. Processing the 3′-Phosphoglycolate Termini

The direct scission of phosphodiester linkages in DNA by bleomycin results in the formation of 3′-phosphoglycolate termini at the site of cleavage. The repair of these lesions requires the removal of the 3′-phosphoglycolate ends and an array of enzymes has been discovered that possess this 3′-phosphodiesterase activity. These include apurinic/apyrimidinic endonuclease I (APE1) and APE2; tyrosyl-DNA phosphodiesterase 1 (TDP1); human nuclease Artemis; and Aprataxin [125,126]. It is generally thought that these enzymes work together to remove specific subsets of the bleomycin-induced 3′-phosphoglycolates, although the precise details are not fully understood.

Izumi et al. showed that the APE1 was a limiting factor in the repair of bleomycin-induced DNA damage [127]. The repair of bleomycin-damaged DNA by a human cellular extract was shown to be enhanced when supplemented with exogenous APE1. However, while APE1 is highly efficient at processing 3′-phosphoglycolates at gapped DNA, the enzyme was observed to be less efficient at removing phosphoglycolates from a 3′-terminus that is located at a recessed or blunt end; and highly inefficient at 3′-overhangs [128].

APE2 was shown to be able to remove phosphoglycolates from 3′-recessed ends [129,130], although it is not known how it compares with APE1.

The endonuclease activity of Artemis can trim long overhangs that contain 3′-phosphoglycolate ends, however, it appears to be less efficient at processing short overhangs [131,132,133]. Fibroblasts from patients who are deficient in Artemis are more sensitive to bleomycin [133]. Transfection of wild-type Artemis cDNA into the deficient fibroblasts restored its resistance to bleomycin [134].

TDP1 was reported to preferentially remove 3′-phosphoglycolates on single-strand DNA or at double-strand breaks [135,136]. Additionally, processing of phosphoglycolates at 3′-overhangs was observed to be three times more efficient, compared to blunt ends; and 10 times more efficient, compared to recessed ends [137]. This enzyme converts the phosphoglycolate terminus into phosphates, which is then likely to be a substrate for the polynucleotide kinase/phosphatase. Cellular extracts from patients deficient in TDP1 were unable to process phosphoglycolates at single-stranded DNA or 3′-overhangs [138]. *Tdp1^−/−^* mice and *Tdp1^−/−^* chicken DT10 cells were reported to be hypersensitive to bleomycin [139,140].

In summary, it is likely that TDP1 is the main enzyme responsible for removing 3′-phosphoglycolate termini from bleomycin cleaved DNA although other enzymes may also play a role.

### 16.2. Genome-Wide Bleomycin Repair

In our genome-wide experiments with bleomycin in HeLa cells, we examined the repair of 3′-phosphoglycolate termini at gene transcription start sites (TSSs) [100]. We found that repair of bleomycin DNA damage preferentially occurred at actively transcribed genes. The most actively transcribed genes had the highest level of repair, while the least actively transcribed genes had the lowest level of repair that was close to non-transcribed genes. There were also differences in repair that depended on whether the transcribed or non-transcribed strand was analysed.

### 16.3. Single-Strand Break Damage Repair

Bleomycin-generated single-strand breaks and 4′-oxidised abasic sites can be repaired by the base excision repair pathway [15,141]. As described above, repair of single-strand breaks is preceded by the removal of the blocking 3′-phosphoglycolate terminus, probably by TDP1 in human cells. Bleomycin-induced 4′-oxidised abasic sites are efficiently processed by the apurinic/apyrimidinic endonuclease activity of APE1, which incises the deoxyribose backbone 5′ to the abasic site. This generates a nick in the DNA and produces a terminal 5′-phosphate at the 4′-oxidised abasic site. APE1 also recruits DNA polymerase β, which possesses a lyase activity that subsequently removes the 5′-phosphate 4′-oxidised abasic site, generating a single nucleotide gap in the DNA [142,143]. The single nucleotide gap is then filled and ligated by DNA polymerase β and the XRCC1/DNA ligase 3 complex, respectively [144]. Alternatively, the gap-filling process in base excision repair can also be undertaken via the long patch repair pathway.

### 16.4. Repair of Double-Strand Breaks

Double-strand breaks can be repaired via homologous recombination or the non-homologous end joining pathway. Both pathways involve an array of protein factors and are still actively being studied (reviewed in [145,146]). Similar to the single-strand damage repair, repair of the bleomycin-induced double-strand breaks requires the removal of the 3′-phosphoglycolate ends.

### 16.5. Repair and Bleomycin Resistance

It is expected that the DNA repair machineries can contribute to the resistance of DNA damaging agents such as bleomycin [102]. Suppression of DNA repair proteins can confer sensitivity of cells to bleomycin and other DNA damaging agents. Conversely, increased expression of proteins known to be involved in DNA repair have also been shown to increase the resistance of cells. For example, overexpression of APE1 in germ cell tumours was shown to increase resistance of these cells to bleomycin [147].

## 17. Cellular Transport of Bleomycin

Bleomycin is administered intravenously as a mixture (Blenoxane), containing predominantly the A_2_ and B_2_ congeners in metal-free form [47]. Once administered, bleomycin has a terminal half-life in the plasma of approximately 90 min, and 65% of it is excreted in the urine within 24 h [12,148,149]. Cu(II) from blood plasma binds to the intravenous bleomycin, forming bleomycin-Cu(II), which is believed to be the complex that is transported into the cells [15,150]. Once inside the cell, it is proposed that the bleomycin-Cu(II) complex is reduced to bleomycin-Cu(I) by cysteine and glutathione [46,151,152]. The Cu(I) ligand can be displaced by Fe(II) and this results in the active bleomycin-Fe(II) complex [153,154].

Bleomycin is a large molecule and hydrophilic, which makes it difficult to cross the cell membrane. However, studies with cobalt-bound bleomycin have shown that the drug binds to a receptor protein on the plasma membrane and then enters the cell via vesicles or receptor-mediated endocytosis. A 250 kDa protein was identified in Chinese hamster fibroblasts as well as in a human head and neck carcinoma cell line [155,156]. However, this protein has not yet been characterised. The subsequent release of bleomycin from endocytotic vesicles is also not fully understood.

A different transport mechanism was reported from studies using *S. cerevisiae* and fluorescently labelled bleomycin. It was found that the L-carnitine transporter Agp2 was able to carry out the uptake of bleomycin. Also, these studies indicated that bleomycin might share a common transport pathway with spermidine or polyamine [157,158]. The pathway requires two kinases Ptk2 and Sky1 to upregulate the transport. Yeast mutants that lack the carnitine transporter Agp2 as well as kinases Ptk2 and Sky1 were shown to have their bleomycin uptake level significantly decreased. The fate of bleomycin entering cells via the energy-dependent transport pathway hinges on whether it is sequestered into cytoplasmic vacuoles for cellular detoxification. The cytotoxicity of bleomycin is only present when it is not degraded by the vacuoles and can diffuse into the cytosol and ultimately the nucleus for DNA cleavage. The human analogue of the l-carnitine transporter, hCT2, was also identified and demonstrated to be involved in bleomycin uptake. The hCT2 protein is highly expressed in human testicular cells, which might explain the effectiveness of bleomycin in the treatment of testicular cancer. In contrast, resistance to the drug seen in human colon and breast cancer might be attributed to the fact that hCT2 is poorly expressed in those tissues [159].

The ability of cells to accumulate intracellular bleomycin is a factor that may contribute to bleomycin resistance or sensitivity. Certain cell membrane proteins, notably the human high affinity l-carnitine transporter, can modulate the accumulation of and subsequently the sensitivity of tumour cells to bleomycin. It was found that overexpression of the yeast polyamine transporter TPO1 increased resistance to bleomycin [160]. The yeast TPO1 transporter was previously found to be involved in the efflux of polyamines from the cell [161,162].

## 18. Bleomycin Hydrolase

Pulmonary toxicity is the major dose-limiting side effect of cancer treatment with bleomycin. Up to 46% of patients encounter pneumonitis or lung inflammation. Long-term usage can lead to lung fibrosis and up to 3% of patients face fatal consequences from pulmonary toxicity [12,15,163].

Bleomycin-induced lung inflammation is thought to result from the low level of bleomycin hydrolase in lung tissue since it is 5–15 fold lower than other tissues [164]. Bleomycin hydrolase, an aminopeptidase, is an enzyme that biochemically deactivates bleomycin. This enzyme catalyses the hydrolysis of the carboxamide group of the β-aminoalanine moiety and forms deamido bleomycin [165]. Deamido bleomycin exhibited DNA cleavage activity, with similar sequence specificity as bleomycin, but was significantly reduced in its ability to mediate double-strand breaks [85,166]. Additionally, studies have shown that the level of bleomycin hydrolase in the lungs correlated with the susceptibility to pulmonary toxicity [164,167]. It is also known that human skin and lungs have low levels of expression of this enzyme, which correlates with the major side effects of the bleomycin treatment [12]. Expression of the yeast bleomycin hydrolase homologue (known to metabolise bleomycin) in mouse NIH3T3 cells increased its resistance to bleomycin—an effect that was reversed when the cells were treated with the cysteine proteinase inhibitor E-64 [168]. In another study, bleomycin hydrolase was knocked-down by RNAi and HeLa cells became 3.4-fold more sensitive to bleomycin [169]. In addition, the bleomycin hydrolase gene has been identified as a methylated tumour suppressor gene in a hepatocellular carcinoma [170]. 

However, the significance of bleomycin hydrolase in mediating resistance against bleomycin is controversial since other studies have reported that bleomycin hydrolase did not appear to confer resistance in yeast cells [171].

## 19. Bleomycin Analogues

There are a number of naturally occurring bleomycin analogues. Studies have reported differences in the cleavage profiles of bleomycin and its naturally occurring analogues, such as talisomycin and phleomycin [56,57]. Notably, the cleavage profile of talisomycin included enhancement for 5′-GA*-3′ sites, which were rarely cleaved by bleomycin [57].

Bleomycin is a complex molecule that makes it difficult to conduct structure-activity studies and there have been relatively few bleomycin analogues produced and tested compared with other clinically-used anti-cancer agents.

Several bleomycin analogues have been produced by altering the His, Ala, Thr, and Cys (bithiazole) amino acids (Figure 1) [14,172]. Alterations to the His and Thr residues resulted in bleomycin analogues with decreased DNA cleavage. However, a phenyl or isopropyl replacement of the methyl group in Ala and a chlorinated bithiazole, produced bleomycin analogues with enhanced DNA cleavage.

The same research group was also able to alter the carbohydrate region of bleomycin A5 and produced three analogues with differences in the carbohydrate region [173]. They found that changes in the carbohydrate region led to major changes in the DNA cleaving ability of the bleomycin analogue. Shen et al. have also found that alterations in the carbohydrate region gave rise to large changes in the DNA cleaving ability of a bleomycin analogue [29].

The successful replacement of a methyl group with a bulky phenyl or isopropyl group at the Ala residue would indicate that other hydrophobic bulky substituents may produce more effective bleomycin analogues. There are also grounds to suspect that bulky group additions to the linker Thr may also lead to more effective analogues [172]. Chlorination of the bithiazole produced interesting bleomycin analogues and hence manipulations of this part of the molecule may also prove productive. In addition, alterations to the disaccharide region resulted in analogues with altered properties and hence this region of the molecule would be a fruitful area to investigate. In particular the replacement of a hydroxyl group with a hydrogen atom resulted in a large change in the DNA cleaving ability of the bleomycin analogue [29].

Bleomycin is natively produced in *S. verticillis* from a large 120 kb gene cluster. However, *S. verticillus* is refractory to transformation, and DNA manipulation and recombinant DNA techniques have proved to be extremely difficult in this bacterium [5]. To avoid this problem, Shen and co-workers have utilised the closely-related bacterium *Streptomyces flavoviridis* to manipulate and express the bleomycin gene cluster [29]. Bleomycin is synthesised using non-ribosomal peptide synthetases and polyketide synthetases from a large 120 kb gene cluster [2,3,4,5,6]. On expressing this 120 kb bleomycin gene cluster in *S. flavoviridis*, three bleomycin analogues, zorbamycin (ZBM), BLM Z, and 6′-deoxy-BLM Z were produced and purified [29].

**Figure 6:**
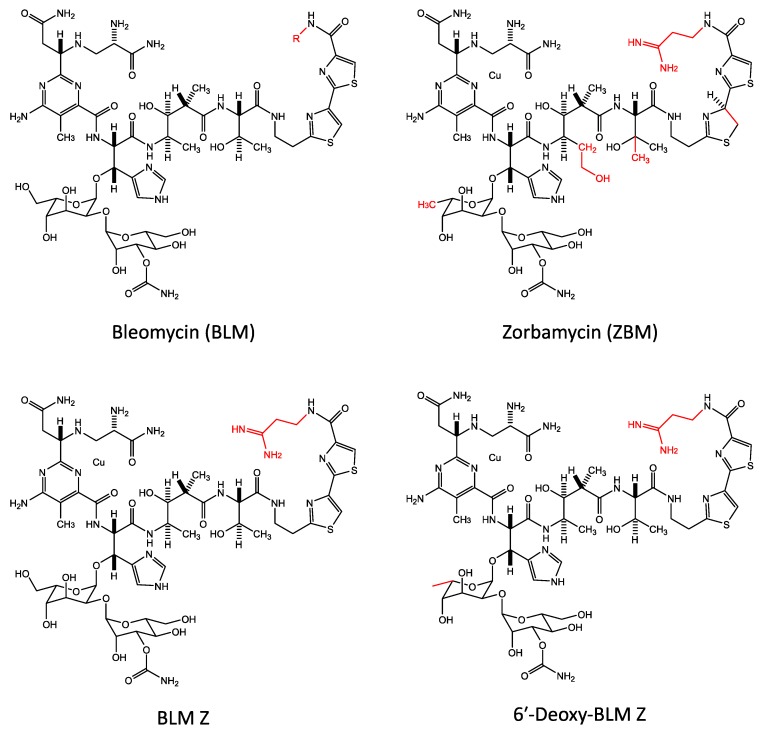
The chemical structures of bleomycin, 6′-deoxy-BLM Z, BLM Z, and ZBM. Differences in chemical structures are shown in red.

The structures of bleomycin and the three bleomycin analogues are shown in Figure 6 and they differ at a small number of positions shown in red. On expressing the bleomycin gene cluster in *S. flavoviridis*, the C-terminal tail is derived from the *S. flavoviridis* biosynthetic apparatus. Thus, the BLM Z, and 6′-deoxy-BLM Z C-terminal tails are similar to ZBM [29]. The structure of bleomycin and BLM Z are exactly the same apart from the C terminal tail (Figure 6).

These three bleomycin analogues that were produced in *S. flavoviridis* were tested for their ability to cleave purified plasmid DNA and it was found that 6′-deoxy-BLM Z was the most efficient at DNA cleavage, followed by ZBM, BLM Z, and bleomycin [29].

The DNA sequence specificity of these three bleomycin analogues was also investigated and it was observed that bleomycin, BLM Z, and 6′-deoxy-BLM Z were very similar, but in comparison, ZBM had a different sequence specificity profile [174]. Bleomycin, BLM Z, and 6′-deoxy-BLM Z were found to mainly cleave at 5′-TGT*A sequences; while the cleavage preference of ZBM was 5′-TGT*G and 5′-TGT*A [174].

Using human HeLa cells, the cytotoxicity was examined and the IC_50_ was 2.9 μM for 6′-deoxy-BLM Z, 3.2 μM for BLM Z, 4.4 μM for bleomycin, and 7.9 μM for ZBM [101].

The genome-wide DNA sequence specificity of 6′-deoxy-BLM Z and ZBM was determined in human HeLa cells and compared with bleomycin [175]. More than 200 million double-strand breaks were analysed for each analogue. For 6′-deoxy-BLM Z, the individual nucleotide consensus sequence was 5′-GTGY*MC (where M is A or C); it was 5′-GTGY*MCA for ZBM; and 5′-GTGT*AC for bleomycin. The most highly ranked tetranucleotides were 5′-TGC*C and 5′-TGT*A for 6′-deoxy-BLM Z; 5′-TGC*C, 5′-TGT*A, and 5′-TGC*A for ZBM; and 5′-TGT*A for bleomycin. Hence, 6′-deoxy-BLM Z and ZBM had a preference for 5′-GC* and 5′-GT* dinucleotides, while it was 5′-GT* for bleomycin in human cellular DNA.

In experiments with purified human genomic DNA, the individual nucleotide consensus sequence was 5′-TGT*A for 6′-deoxy-BLM, 5′-RTGY*AYR for ZBM, and 5′-TGT*A for bleomycin. Thus, the purified genome-wide DNA sequence specificity was similar for bleomycin and 6′-deoxy-BLM, but was different for ZBM. In addition, the cellular DNA sequence specificities for the analogues, were different in cellular DNA compared with purified DNA. As mentioned above, there are many differences in the cellular environment compared with purified DNA, for example, chromatin structure. The differences in sequence specificity between the two environments are greater for the two analogues compared with bleomycin. Hence, the analogues must be more sensitive to these differences than bleomycin. It also shows that caution should be applied when extrapolating results with purified DNA to cellular DNA.

We also examined the effect of chromatin structure on the cellular DNA cleavage of the two analogues in comparison with bleomycin [101]. As for bleomycin, it was found that 6′-deoxy-BLM Z and ZBM preferentially cleaved at the transcription start sites (TSSs) of actively transcribed genes in human cells. The extent of preferential cleavage at the TSSs was quantified and it was observed to correlate with the cytotoxicity of the bleomycin analogues. This preferential cleavage at the TSSs is consistent with the concept that DNA double-strand breaks are the crucial lesion for the cytotoxicity of bleomycin.

As found for bleomycin, 6′-deoxy-BLM Z and ZBM cleaved in the linker region of the nucleosome [99,100,101]. These analogues were also able to detect positioned nucleosomes at the TSSs in human cells [99,101].

## 20. Production of Novel Bleomycin Analogues That Are Resistant to Cleavage by Bleomycin Hydrolase

The modular structure of non-ribosomal peptide synthetases and polyketide synthetases on the 120 kb gene cluster enables facile modification and manipulation of this cluster to produce novel bleomycin analogues [2,3,4,5,6]. Hence, the selective engineering of specific modules in the bleomycin synthetic pathway via combinatorial biosynthesis [29] opens the door for efficient production of novel bleomycin analogues that could have beneficial cancer chemotherapeutic properties.

As mentioned above, a major concern in the administration of bleomycin lies in its dose-limiting side effect, pulmonary toxicity, and up to 46% of patients encounter this side effect [12]. This toxicity is the major limitation for therapy and hence, it has been of interest to develop more effective analogues that can overcome this limitation. As mentioned above, bleomycin is inactivated by the endogenous enzyme bleomycin hydrolase and this enzyme is found at low levels in lung tissue. The production of bleomycin analogues that are not cleavable by human bleomycin hydrolase would result in lung tissue being as equally susceptible to bleomycin activity as other tissues. Hence this bleomycin analogue would be more effective as an anti-cancer agent because the selective lung toxicity has been eliminated. Resistance to bleomycin in other tumour cell types via the over-expression of bleomycin hydrolase would also be bypassed. 

The bleomycin hydrolase cleaves the amide bond in the β-aminoalanine moiety (green arrow in Figure 1). Manipulation of this part of the molecule could achieve the desired bleomycin analogue; namely an analogue with DNA cleaving ability but refractory to cleavage by bleomycin hydrolase. Other strategies, for example, the attachment of a large lipophilic group [176], may also prove viable.

There have been a number of studies where bleomycin analogues have been chemically synthesised [172]. However, none of these studies examined whether bleomycin hydrolase could cleave the bleomycin analogues.

## 21. Summary and Future Prospects

Bleomycin is a complex molecule and unlike other smaller anti-cancer agents, has not been extensively modified to conduct structure-activity studies.

There are several possible approaches to establish a more effective bleomycin analogue.

1. One approach is to produce bleomycin analogues that are resistant to cleavage by bleomycin hydrolase. The anti-tumour activity of bleomycin is limited by lung toxicity. The production of bleomycin analogues that are not cleaved by human bleomycin hydrolase will result in bleomycin analogues that are more effective as an anti-cancer agent because the lung toxicity would be eliminated.
2. The engineering of an analogue that has improved uptake into cells, or even better, preferential uptake into tumour cells would produce a more effective cancer chemotherapeutic agent. Alterations to the disaccharide region could be prime areas for modification to achieve this aim.
3. The production of analogues with faster/greater binding to DNA would target the analogue to the biological target DNA and eliminate side reaction with other molecules—the DNA targeting hypothesis [177,178]. The bithiazole tail could be modified to produce greater DNA binding.
4. The creation of an analogue that is more efficient in producing the “activated” intermediate could have beneficial properties.
5. More complicated and problematic would be the engineering of an analogue that is more effective at producing double-strand breaks compared with single-strand breaks, since double-strand breaks are thought to be the crucial lesion for the cytotoxicity of bleomycin.
6. Further investigations with genome-wide studies will determine the crucial genes that are preferentially cleaved by bleomycin. In combination with nucleic acid-based techniques that target these crucial genes, bleomycin cytotoxicity could be enhanced by focusing on these important genes.
7. Synergies could also be found with other nucleic acid and antibody-based novel therapies to enhance the action of these recently introduced therapeutics.

The clinical importance of bleomycin as a cancer chemotherapeutic agent suggests that improved analogues based on bleomycin can be developed. Experiments that provide a deeper molecular understanding of the crucial constituents of the bleomycin molecule could lead to directed synthesis of highly effective bleomycin analogues. Tools exist that provide a quantitative and precise platform whereby bleomycin analogues can be tested and compared. This information, along with knowledge of their structure and their efficacy, could provide an informed basis for the development of new more efficient anti-cancer analogues based on bleomycin.

## Figures and Tables

**Table 1:** The preferred nucleotides at the bleomycin cleavage site.

Study	Type of Break	Preferred Individual Nucleotides						Consensus Sequence from the Individual Nucleotide Data	Consensus Sequence from Complete Sequence Data
Position		−3	−2	−1	0*	+1	+2		
Early ^32^P-end-label experiments	SSB		T	G	T			5′-TGT*	
Random DNA sequence	SSB		T	G	T	A		5′-TGT*A	5′-TGT*A
Systematically altered RTGTAY clone	SSB	C > T	C = T	G	T	A	T = A	5′-YYGT*AW	
Purified DNA genome-wide preferred nucleotide (50k)	DSB	ns	T	G	T	A	T > A	5′-TGT*AW	5′-TGT*AT
Cellular DNA genome-wide preferred nucleotide (50k)	DSB	G	T	G	T	A	ns	5′-GTGT*A	5′-RTGT*AY

The preferred nucleotides at the bleomycin cleavage site are shown from the −3 to the +2 positions. Only the most highly preferred nucleotides are shown. The preferred nucleotides from the genome-wide data for cellular and purified human DNA are depicted for the top 50,000 cleavage sites. Note that the individual nucleotide analysis at the bleomycin cleavage site (second method), is shown in this Table except for the column on the right where the complete sequence (first method) data is shown. SSB—single-strand break; DSB—double-strand break; ns—not significant. The * indicates the nucleotide at position 0 that is cleaved and destroyed during the reaction.

## Abbreviations

APE1 and APE2	Apurinic/apyrimidinic endonuclease 1 and 2
CE-LIF	Capillary electrophoresis with laser-induced fluorescence detection
TSS	Transcription start site
TDP1	Tyrosyl-DNA phosphodiesterase 1
ZBM	zorbamycin
M	A or C nucleotides
R	G or A nucleotides
W	A or T nucleotides
Y	T or C nucleotides
